# How to Reorganize the Radiology Departments to Face the 2019 Coronavirus Disease Outbreak

**DOI:** 10.1017/dmp.2020.159

**Published:** 2020-05-19

**Authors:** Michaela Cellina, Marcello Orsi, Giancarlo Oliva

**Affiliations:** Department of Radiology, Ospedale Fatebenefratelli, ASST Fatebenefratelli Sacco, Milan, Italy

**Keywords:** COVID-19, pneumonia, viral, radiology department, hospital, severe acute respiratory syndrome coronavirus 2, tomography, spiral computed

## Abstract

Radiology departments have been directly involved from the beginning of the novel coronavirus disease (COVID-19) emergency to provide imaging lung assessment of suspected and positive patients while ensuring the execution of other routine and emergency examinations for non-COVID-19 patients. To limit the risk of the infection spread, radiology departments should be reconfigured. We propose the example of the reorganization of the Radiology Department of our hospital, in the center of Milan, in Northern Italy, which consisted of the creation of 2 completely distinct pathways and distinct radiological machines for COVID-19 positive or suspected positive and for non-COVID-19 patients.

The novel coronavirus disease (COVID-19) was first reported in Wuhan, Hubei Province, China, in December 2019.^1^ It spread rapidly worldwide with more than 3.5 million confirmed cases and more than 200 countries involved on May 6, based on the World Health Organization situation report-107.^2^ Because the most common clinical manifestation consists of pneumonia, radiology departments have been directly involved from the beginning of this emergency, to assess lung parenchyma abnormalities with computed tomography (CT) or X-ray in positive or suspected positive COVID-19. However, radiology departments must also ensure election and urgency examinations for COVID-19 negative patients, while guaranteeing their safety. Reconfiguration of the radiology units is therefore required to prevent further transmission of the virus to patients and staff members. We reported the reorganization of the Radiology Department that we established in our hospital, in the center of Milan.

## STAFF TRAINING

Just after the COVID-19 outbreak, comprehensive training for the whole hospital staff about procedures and safety measures was performed with on-site small group courses and online didactic materials.^3^ At the end of each course, a verification test was carried out to certify correct learning.

The training content includes hand hygiene, correct use of personal protective equipment (PPE), the sequence for donning and doffing PPE,^4^ different pathways and management for suspected and non-suspected patients, isolation measures, the disposal of used PPE and medical waste, the environment, and surface cleaning and disinfection procedures.

### Personal Protective Equipment

Radiology technologists (RTs) are among the first-line health care workers who might be exposed to COVID-19; therefore, they should be particularly careful in the use of adequate precautions.

COVID-19 spreads through droplet and contact transmission. When dealing with suspected patients, the PPE to protect from contact, droplet, and airborne transmission is needed. The PPE set includes eye protection (goggles or face shield) to prevent virus exposure of the eye mucosa, a filtering face-piece respirator (FFP2), a surgical cap, a double pair of disposable gloves (inner and outer layer), a disposable long-sleeved fluid-resistant gown, and shoe covers.

Before putting on PPE, proper hand hygiene should be performed.^5^ Alcohol-based hand gels for hand hygiene dispensers are available in all dressing rooms, reading rooms, and diagnostic units. The wearing and removal of PPE are performed in 2 different dedicated rooms. The PPE wearing takes place in a room with direct access to the COVID-19 Radiology and includes the following items in sequence: disposable gown, surgical cap, protective FFP2 mask (fitting the metal nose clip), goggles over the mask straps, inner latex gloves, outer latex gloves layer, covering over the gown’s cuff, and shoe covers.^6^ A second operator helps in closing the gown back and checks that the dressing procedure is carried out correctly.

The proper PPE removal sequence should be performed to avoid self-contamination. The cleaning of visible dirt, if present, followed by hand hygiene with alcohol-based disinfectant, the removal of shoe covers, followed by hand hygiene, and removal of the outer pairs of gloves, followed by hand hygiene take place in the CT or X-ray unit rooms^7^; the used PPE items are put in biohazard containers for special medical waste.

The second part of decontamination and removal of PPE take place in a second dedicated room with direct access from COVID-19 Radiology and involve a similar sequence of steps: taking off the disposable gown with the help of an assistant, hand hygiene, removal of the goggles or face shield, hand hygiene, taking off medical protective masks, hand hygiene, taking off the cap and of inner gloves, and final hand-washing.^6^ A second operator helps the first one in the gown removal and checks that the undressing steps are carried out correctly.

Because a certain division between truly positive and negative cases at patient arrival is impossible due to the presence of COVID-19 positive but asymptomatic patients, and considering the long incubation period of the disease in both radiology units, the staff should consider all of the patients as potentially COVID-19 positive until proven otherwise and, therefore, use the highest level available of PPE against droplets and contact transmission.

### Patients’ Entrance

Checkpoints for temperature measurements of all outpatients and staff have been set at the entrances of the hospital. Signs to remember the respect of the safety distance and the mandatory use of surgical masks were hung at the entrances and in all waiting rooms.

### Creation of Distinct Routes and Radiology Unit Reconfiguration

As already suggested,^7,8^ the creation of 2 separate pathways for COVID-19 positive (and suspected positive) and COVID-19 negative patients represents the best solution to avoid the spread of the infection. We divided our Radiology Department into a dedicated COVID-19 radiology unit and a COVID-19-free radiology unit.

The division of patients begins in the triage area of the emergency department (ED), where trained staff aim to select subjects with high or low probability to have COVID-19 infection, according to their symptoms, clinical parameters, and history of exposure. Therefore, 2 distinct waiting areas for patients with and without respiratory symptoms and fever have been created.

Regardless of the level of suspicion, each patient receives a surgical face mask at entrances of the ED and the Radiology Department.

From the ED, health care assistants with appropriate PPE bring patients to perform the required radiological examinations in the 2 radiology units with distinct paths, according to the assigned risk. The 2 paths are indicated by signs on the wall and are separated by barriers.

The COVID-19 Radiology Unit is located in the basement of the building where the ED and intensive and subintensive COVID-19 units are located and accessible from a dedicated elevator.^9^ This unit includes a CT scanner, 2 X-ray machines, 1 ultrasound machine, and rooms dedicated to personnel, 1 for wearing the PPE and 1 for decontamination, with removing PPE items, which are put into special waste bins. These were originally reading rooms that have been converted.

According to the American College of Radiology recommendations,^10^ portable radiography machines have been positioned in the ED and in COVID-19 units to perform chest X-rays when a lung evaluation is medically needed, reducing patient handling risks. The activity should work 24/7; therefore, a reorganization of the radiological staff is needed.

For the execution of chest CT, the presence of 2 RTs represents the best solution^11,12^: 1 RT with PPE sets up the patient positioning on the scanner table, whereas a second RT with a surgical mask and gloves operates the machine console. Disposable surgical sheets are employed to cover the CT scanner table to avoid contacts between the patient and the CT equipment. In contrast-enhanced CT, the medical radiologist and nurse wear PPE for direct assistance to the patient.

Two RTs are dedicated to the execution of X-rays: 1 wears PPE for direct assistance, for positioning the patient or the X-ray cassette behind the patient’s back and to center the patient; and the other RT, equipped with a surgical mask and gloves, sets the machine (exposure data), delivers X-rays, and checks the images to be sent to Picture Archiving and Communication System (PACS).

The non-COVID-19 patients admitted in the ED have a separate route with access to the clean Radiology Unit, dedicated also to non-COVID-19 inpatients and outpatients.

### Disinfection Procedures

All RTs and health care assistants have also been trained adequately on the disinfection procedures that need to be applied to each machine. After each examination, the CT suite is closed for the next hour to exchange the room air and to allow a specifically trained health care assistant, protected with PPE, to perform the removal of the disposable sheet and the cleaning of the CT gantry and all contact surfaces with a cloth soaked with alcohol-based disinfectants (75% ethanol). Disinfection of the radiography equipment is performed between uses by RTs (protected with PPE) wiping the surface using 75% ethanol. After each examination, the X-ray equipment, the contact surfaces of the CT gantry and table, and CT console rooms are sanitized with alcohol-based disinfectants.

## CONCLUSION

It is likely that radiology departments will always have a key role in the diagnosis and follow-up of infectious diseases and in the infection control, on account of a timely imaging diagnosis.^13,14^ The experience gained in this emergency must not only help establish a rapid reconfiguration of the units, when needed, to minimize in-hospital transmission, but also spread a culture of infection control practice to the radiological and the whole hospital staff. The collaboration among the staff is essential to get the right safety measures within the Radiology Department and the whole hospital. Development and continuous implementation of strict procedures can provide protection against the transmission of any viral agent.

The reorganization of our Radiology Department, with the creation of 2 distinct pathways, could be a possible way to face the COVID-19 outbreak. Dedicated radiology units and dedicated radiology equipment can reduce the risk of infection transmission and contribute in the successful management of this emergency.

## Abbreviations

COVID-19: novel coronavirus disease
CT: computed tomography
ED: emergency department
FFP2: filtering face-piece respirator
PPE: personal protective equipment
RT: radiology technologist
